# Resisting and tolerating *P. falciparum* in pregnancy under different malaria transmission intensities

**DOI:** 10.1186/s12916-017-0893-6

**Published:** 2017-07-17

**Authors:** Nicaise Tuikue Ndam, Emmanuel Mbuba, Raquel González, Pau Cisteró, Simon Kariuki, Esperança Sevene, María Rupérez, Ana Maria Fonseca, Anifa Vala, Sonia Maculuve, Alfons Jiménez, Llorenç Quintó, Peter Ouma, Michael Ramharter, John J. Aponte, Arsenio Nhacolo, Achille Massougbodji, Valerie Briand, Peter G. Kremsner, Ghyslain Mombo-Ngoma, Meghna Desai, Eusebio Macete, Michel Cot, Clara Menéndez, Alfredo Mayor

**Affiliations:** 1 Institut de Recherche pour le Développement (IRD), Paris, France; 2 COMUE Sorbonne Paris Cité, Faculté de Pharmacie, Paris, France; 3 Faculté des Sciences de la Santé (FSS), Université d’Aboméy Calavi, Cotonou, Benin; 4 Ifakara Health Institute (IHI), Bagamoyo Research and Training Centre (BRTC), Bagamoyo, Tanzania; 5 ISGlobal, Barcelona Ctr. Int. Health Res. (CRESIB), Hospital Clínic - Universitat de Barcelona, Barcelona, Spain; 6 Centro de Investigação em Saúde da Manhiça (CISM), Maputo, Mozambique; 7 Universidade Eduardo Mondlane, Maputo, Mozambique; 8 Kenya Medical Research Institute (KEMRI)/Centre for Global Health Research, Kisumu, Kenya; 9 Graduate Program in Areas of Basic and Applied Biology, Universidade do Porto, Porto, Portugal; 10 CIBER Epidemiología y Salud Pública (CIBERESP), Madrid, Spain; 11 Institute of Tropical Medicine, University of Tübingen, Tübingen, Germany; 12 Department of Medicine I, Division of Infectious Diseases and Tropical Medicine, Medical University of Vienna, Vienna, Austria; 13 Centre de Recherches Médicales de Lambaréné (CERMEL), Lambaréné, Gabon; 14 Malaria Branch, Division of Parasitic Diseases and Malaria, Center for Global Health, Centers for Disease Control and Prevention, Atlanta, Georgia USA

**Keywords:** Malaria, Pregnancy, Immunity, Resistance, Tolerance

## Abstract

**Background:**

Resistance and tolerance to *Plasmodium falciparum* can determine the progression of malaria disease. However, quantitative evidence of tolerance is still limited. We investigated variations in the adverse impact of *P. falciparum* infections among African pregnant women under different intensities of malaria transmission.

**Methods:**

*P. falciparum* at delivery was assessed by microscopy, quantitative PCR (qPCR) and placental histology in 946 HIV-uninfected and 768 HIV-infected pregnant women from Benin, Gabon, Kenya and Mozambique. Resistance was defined by the proportion of submicroscopic infections and the levels of anti-parasite antibodies quantified by Luminex, and tolerance by the relationship of pregnancy outcomes with parasite densities at delivery.

**Results:**

*P. falciparum* prevalence by qPCR in peripheral and/or placental blood of HIV-uninfected Mozambican, Gabonese and Beninese women at delivery was 6% (21/340), 11% (28/257) and 41% (143/349), respectively. The proportion of peripheral submicroscopic infections was higher in Benin (83%) than in Mozambique (60%) and Gabon (55%; *P* = 0.033). Past or chronic placental *P. falciparum* infection was associated with an increased risk of preterm birth in Mozambican newborns (OR = 7.05, 95% CI 1.79 to 27.82). Microscopic infections were associated with reductions in haemoglobin levels at delivery among Mozambican women (–1.17 g/dL, 95% CI –2.09 to –0.24) as well as with larger drops in haemoglobin levels from recruitment to delivery in Mozambican (–1.66 g/dL, 95% CI –2.68 to –0.64) and Gabonese (–0.91 g/dL, 95% CI –1.79 to –0.02) women. Doubling qPCR-peripheral parasite densities in Mozambican women were associated with decreases in haemoglobin levels at delivery (–0.16 g/dL, 95% CI –0.29 to –0.02) and increases in the drop of haemoglobin levels (–0.29 g/dL, 95% CI –0.44 to –0.14). Beninese women had higher anti-parasite IgGs than Mozambican women (*P* < 0.001). No difference was found in the proportion of submicroscopic infections nor in the adverse impact of *P. falciparum* infections in HIV-infected women from Kenya (*P. falciparum* prevalence by qPCR: 9%, 32/351) and Mozambique (4%, 15/417).

**Conclusions:**

The lowest levels of resistance and tolerance in pregnant women from areas of low malaria transmission were accompanied by the largest adverse impact of *P. falciparum* infections. Exposure-dependent mechanisms developed by pregnant women to resist the infection and minimise pathology can reduce malaria-related adverse outcomes. Distinguishing both types of defences is important to understand how reductions in transmission can affect malaria disease.

**Trial registration:**

ClinicalTrials.gov NCT00811421. Registered 18 December 2008.

**Electronic supplementary material:**

The online version of this article (doi:10.1186/s12916-017-0893-6) contains supplementary material, which is available to authorized users.

## Background

As the rest of the population, pregnant women living in areas of different malaria endemicity experience varying degrees of exposure to infection, which affects the acquisition of antimalarial immunity and determines the course of disease [1, 2]. Protective immunity to *Plasmodium falciparum* in pregnancy has been suggested to rely mostly on antibodies against VAR2CSA that block adhesion of infected erythrocytes to placental chondroitin sulphate A, and thereby prevent parasite sequestration in the placenta [3]. Such immune resistance to *P. falciparum*, which is acquired after exposure to placental-type parasites, reduces parasite densities below the detection limit of microscopy (i.e. submicroscopic infections) [4–6] and can eventually clear placental infections.

Infected hosts may also tolerate the presence of *P. falciparum* by minimising parasite-induced damage without necessarily limiting the infection [7–9]. This type of host defence, not to be confused with immunological tolerance [7, 10], has been suggested by the frequent observation in malaria endemic areas of individuals, including pregnant women, who harbour levels of parasitaemia in their blood that would commonly be associated with fever in malaria-naïve individuals [11, 12]. Moreover, the higher risk of life-threatening disease in younger age groups [13] supports the notion that the ability to modulate host inflammation (anti-disease or clinical immunity) [14, 15] develops faster than the capacity to restrict parasite growth (anti-parasite immunity). However, other studies do not support the hypothesis that a special clinical immunity exists independently of parasitological immunity [16], but rather suggest that immunity resulting in decreased parasite densities reduces the severity of symptoms.

Resolving the role of resistance and tolerance could aid the development of host-directed therapies to reduce malaria-induced immunopathology and mitigate malaria disease [9]. However, quantitative analyses of tolerance to human malaria have been mainly limited to the assessment of peripheral *P. falciparum* parasitemia needed to trigger the onset of fever (i.e. pyrogenic threshold) [14, 15]. Alternative clinical outcomes and analytical frameworks are needed for pregnant women in whom parasitaemia is poorly associated with fever [17]. Here, we aimed to assess the variations in the clinical impact of *P. falciparum* infections and in host defences developed by pregnant women under different malaria transmission intensities. To achieve this, we compared the carriage of submicroscopic infections and antibodies against *P. falciparum* antigens as indicators of the level of parasitological immunity [1]. We assessed the correlation between health outcomes (haemoglobin levels and birthweight) and parasite densities at delivery for summarising tolerance [8], with a flat slope indicative of tolerance to infection. As immune resistance is strongly influenced by the number of previous pregnancies in areas of stable transmission [18] and pregnancy outcomes can be affected by the preventive measures used during pregnancy, analyses were adjusted for parity of the pregnant women and the antimalarials received as intermittent preventive treatment during pregnancy (IPTp).

## Methods

### Study populations

This study was conducted between 2010 and 2012 in four sub-Saharan countries (Additional file 1: Figure S1), namely Benin (Allada, Sékou and Attogon), Gabon (Lambaréné and Fougamou), Kenya (Siaya), and Mozambique (Manhiça and Maragra). Pregnant women were enrolled in the context of the Malaria in Pregnancy Preventive Alternative Drugs clinical trial (ClinicalTrials.gov NCT0081121; Table 1) [19, 20]. At enrolment, pregnant women received long-lasting insecticide-treated bed-nets. Following national guidelines in place, HIV status was assessed after voluntary HIV counselling and testing with an HIV rapid test and positive results were confirmed with a second rapid test [19, 20]. Haemoglobin and the syphilis rapid plasma reagin (RPR) test were assessed as part of routine antenatal care on finger-prick-collected capillary blood. Among HIV-infected women, 5 mL of venous blood were taken for CD4 + T cell count by flow cytometry after staining of whole blood with CD3, CD8 and CD4 fluorochrome-labelled antibodies. Flow cytometry acquisition was performed using FACSCalibur (BD Biosciences) and TruCOUNT tubes (Becton Dickinson). Additionally, viral load determination was performed using the COBAS AMPLICOR or AmpliPrep (Roche Diagnostics) devices. Haemoglobin was determined in capillary blood samples using mobile devices (HemoCue and Hemocontrol) [19, 20]. Gestational age at enrolment was determined from symphysis fundal height measurement using a standard tape measure (centimetres) and McDonald’s rule to transform the symphysis fundal height in centimetres into gestational weeks [19, 20]. Following physical examination, recruited women with gestational age ≥ 13 weeks received their first dose of IPTp (either sulfadoxine-pyrimethamine (SP) or mefloquine (MQ) for HIV-uninfected women and either placebo or MQ for HIV-infected women) under supervision. Women allocated to the SP group received standard IPTp (three tablets of the fixed combination therapy containing 500 mg of sulfadoxine and 25 mg of pyrimethamine), whereas participants allocated to the MQ groups received 15 mg/kg of the drug. The second IPTp-SP/MQ administration for HIV-uninfected women, and the second and third administrations of IPTp-MQ/placebo for HIV-infected women, were given at least 1 month apart. All HIV-infected women also received study co-trimoxazole tablets on a monthly basis for daily prophylaxis.

**Table 1:** Characteristics of participants by study area

	HIV-uninfected^a^				HIV-infected^b^		
	All	Benin	Gabon	Mozambique	All	Kenya	Mozambique
	n = 946	n = 349	n = 257	n = 340	n = 768	n = 351	n = 417
Age (years), mean (SD)							
	24.3 (6.3)	25.4 (5.2)	24.4 (6.7)	23.1 (6.8)	26.7 (5.7)	26.9 (5.4)	26.5 (5.9)
Parity, n (%)							
Primigravidae	265 (28)	69 (20)	66 (26)	130 (38)	81 (11)	30 (9)	51 (12)
Multigravidae	681 (72)	280 (80)	191 (74)	210 (62)	687 (89)	321 (91)	366 (88)
Gestational age at baseline (weeks), mean (SD)							
	21.0 (4.9)	22.0 (4.1)	19.5 (5.4)	21.3 (5.2)	20.3 (5.5)	19.9 (5.9)	20.6 (5.2)
MUAC at baseline (cm), mean (SD)							
	25.9 (3.0)	25.3 (2.3)	25.7 (3.6)	26.8 (3.0)	26.8 (2.7)	27.0 (2.7)	26.7 (2.7)
RPR at baseline, n (%)							
Positive	9 (1)	2 (1)	1 (0)	6 (2)	39 (5)	12 (3)	27 (7)
Negative	928 (99)	347 (99)	247 (100)	334 (98)	726 (95)	338 (97)	388 (93)
Literate, n (%)							
No	371 (39)	280 (80)	34 (13)	57 (17)	142 (18)	16 (5)	126 (30)
Yes	575 (61)	69 (20)	223 (87)	283 (83)	626 (82)	335 (95)	291 (70)
HIV viral load (copies/mL) at baseline, n (%)^c^							
< 399					181 (24)	125 (36)	56 (13)
400/999					183 (24)	76 (22)	107 (26)
1000/9999					243 (32)	97 (28	146 (35)
10,000/max					123 (16)	49 (14)	74 (18)
UNK					38 (5)	4 (1)	34 (8)
CD4 (cells/μL) at baseline, n (%)^d^							
< 350					279 (36)	137 (39)	142 (34)
350/max					460 (60)	212 (60)	248 (59)
UNK					29 (4)	2 (1)	27 (6)
Maternal haemoglobin at baseline (g/dL), mean (SD)							
	10.4 (1.5)	10.3 (1.2)	10.3 (1.4)	10.7 (1.8)	10.1 (1.7)	10.2 (1.9)	10.0 (1.6)
IPTp, n (%)							
Antimalarial 1^e^	629 (66)	230 (66)	172 (67)	227 (67)	390 (51)	174 (50)	216 (52)
Antimalarial 2^e^	317 (34)	119 (34)	85 (33)	113 (33)	378 (49)	177 (50)	201 (48))
Gestational age at delivery (weeks),^f^ mean (SD)							
	39.6 (1.7)	39.2 (1.2)	41.1 (1.8)	38.9 (1.2)	38.8 (1.2)	ND	38.8 (1.2)
Preterm birth,^g^ n (%)							
No	867 (95)	328 (95)	238 (95)	301 (95)	374 (96)	ND	374 (96)
Yes	44 (5)	16 (5)	13 (5)	15 (5)	17 (4)	ND	17 (4)

^a^The IPTp trial evaluated the efficacy and safety of two doses of Intermittent Preventive Treatment in pregnancy (IPTp) with mefloquine compared to IPTp with sulphadoxine-pyrimethamine

^b^Evaluated three doses of IPTp with MQ compared to placebo in HIV-infected women on cotrimoxazole prophylaxis

^c^38 missing values

^d^29 missing values

^e^Antimalarial: 1 = Mefloquine and 2 = Sulphadoxine-Pyrimethamine for HIV-uninfected; 1 = Placebo and 2 = Mefloquine for HIV-infected

^f^Gestational age was estimated by newborn physical examination using the Ballard score

^g^Preterm birth if gestational age was < 37 weeks

*IPTp* intermittent preventive treatment, *MUAC* mid-upper arm circumference, *RPR* rapid plasma reagin, *UNK* unknown

At delivery, maternal haemoglobin was determined and newborns were weighed using weekly calibrated scales (either digital or three beam balances) and their gestational age at birth (except for Kenyan newborns) was evaluated using the Ballard’s score [21]. Newborn weights not captured at birth but within the first week of life were estimated using a linear regression model [22]. Peripheral and placental blood smears, as well as 50 μL of maternal peripheral and placental blood spotted onto filter paper, were collected for parasitological assessments. Tissue samples were also collected from the maternal side of the placenta and placed into 10% neutral buffered formalin. Biopsies were processed, stained and examined following standard procedures [23]. From the first antenatal visit, all study women were indicated to receive ferrous sulphate-folic acid supplements for prevention of anaemia in pregnancy. If a woman was diagnosed with anaemia, she was treated with oral ferrous sulphate 200 mg/8 hours for 3 months or blood transfusion for severe cases. Clinical malaria episodes were treated with oral quinine (first trimester) or artemether-lumefantrine (subsequent trimesters) for uncomplicated malaria; parenteral quinine was used for treatment of severe malaria [19, 20].

### Parasitological determinations

Thick and thin blood films, as well as placental biopsies, were read for *Plasmodium* species detection according to standard, quality-controlled procedures [19, 20, 24]. The quality of the reading across sites was controlled through a quality control program during the study. Past placental infection was defined by the presence of *P. falciparum* pigment (i.e. hemozoin) without parasite detection on placental histologic examination, and chronic placental infection was defined by the presence of *P. falciparum* pigment in combination with the detection of parasites [1]. A 30% random selection of paired peripheral and placental blood onto filter papers from HIV-negative women [20] and all the paired filter papers from HIV-infected women [19] were tested for the presence and density of *P. falciparum* in duplicate by means of a real-time quantitative polymerase chain-reaction (qPCR) targeting 18S rDNA [1, 25]. Parasitemia was quantified by extrapolation of cycle thresholds from a standard curve of *P. falciparum* ring-infected erythrocytes. Samples without amplification (no cycle thresholds detected) were considered negative, and a density of 2 parasites/μL was assigned if amplification was observed out of the lower range of the standard curve (5 parasites/μL). A negative control with no template DNA was run in all reactions. *P. falciparum* infections were considered submicroscopic if parasites were detected by qPCR but not by microscopy [1]. A quality check program was established to ensure comparable performance of qPCR techniques in different laboratories [25].

### Measurement of antimalarial IgGs and *P. falciparum* Histidine-Rich Protein 2 (PfHRP2)

A random selection of 50% of peripheral plasma samples collected at delivery from HIV-uninfected women in the extremes of the malaria transmission spectrum (n = 170 from Benin and n = 170 from Mozambique) was tested for IgG levels against *P. falciparum* recombinant VAR2CSA domain (DBL3X from 3D7 strain), and the merozoite surface protein-1 (MSP-1_19_; 19-kD fragment, 3D7 strain) using a multiplex suspension array panel (xMAP^TM^ technology) and the Luminex® 100/200™ System (Luminex Corp., Austin, TX, USA) [1]. Briefly, magnetic carboxylated microspheres (MagPlex^TM^-C, Luminex) were coupled with recombinant protein. After blocking with bovine serum albumin in phosphate-buffered saline, microspheres were sequentially incubated with 100 μL of plasma (dilutions 1:500, 1:20,000 and 1:800,000 in duplicate for each sample), 100 μL of biotinylated anti-human IgG (diluted 1:2500) and 100 μL of streptavidin-conjugated R-phycoerythrin (diluted 1:1000). The plate was immediately read using Bio-Plex Manager version 4.0, and at least 50 microspheres per analyte were acquired per sample. Crude mean fluorescent intensity was exported with background fluorescence from blank wells already subtracted. PfHRP2 [26] was quantified in plasmas available from women with peripheral qPCR-detected *P. falciparum* infection at delivery (n = 42 in Benin and n = 13 in Mozambique) using a commercial HRP2 antigen-capture ELISA (Malaria Ag CELISA kit; Cellabs) [27].

### Statistical analysis

Pregnant women were included in the analysis if they had all information on IPTp type of treatment, HIV infection, age, parity, newborn weight, maternal haemoglobin levels, as well as qPCR results in peripheral and placental blood at time of delivery. Women were classified as primigravid (first pregnancy) and multigravid (at least one previous pregnancy). Age was categorised as < 25 and ≥ 25 years on the basis of median maternal age in the study population. Preterm birth was defined as a gestational age < 37 weeks. Participant’s baseline characteristics, parasitological outcomes, antimalarial IgG levels and PfHRP2 levels were compared between study areas by univariate analysis and logistic or lineal regression models. Changes in maternal haemoglobin levels and birthweight with increases in qPCR-peripheral parasite density were assessed by linear regression. The impact of *P. falciparum* infections on haemoglobin levels, birthweight and preterm birth was assessed in logistic regression models. Continuous outcome variables (qPCR-parasite densities, antimalarial IgGs and HRP2 levels) exhibiting a skewed distribution were log transformed. Regression models were estimated through a backward stepwise approach with a significance level for removal from the model of 0.1 and a significance level for addition of 0.05, adjusted by type of IPTp drug and baseline covariates at recruitment (season, age, gravidity, gestational age, anaemia, literacy, RPR result and mid-upper arm circumference (MUAC)), as well as CD4 + T cell count in the case of HIV-infected women [19, 20]. The modification of the associations by study area was assessed by including interaction terms into the regression models and combining the coefficients plus the interaction and the standard error by the delta method. *P* values of less than 0.05 were considered to indicate statistical significance. Stata version 13 was used for data analysis and GraphPad version 5.01 for graphical depiction of data.

## Results

### *P. falciparum* burden in the study populations

**Fig. 1:**
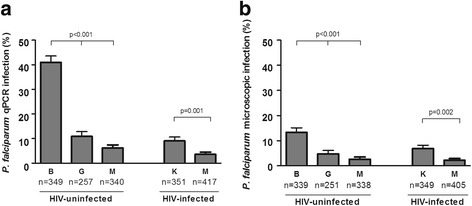
Prevalence of *P. falciparum* maternal infection according to study area. Prevalence of *P. falciparum* at delivery in peripheral and/or placental blood as detected by qPCR (**a**) and microscopy (**b**). *P* value according to the multivariate analysis adjusted for type of IPTp drug, season, age, gravidity, gestational age, anaemia, literacy, RPR result and MUAC at recruitment, as well as CD4 + T cell count in the case of HIV-infected women. T bars represent standard errors. *B* Benin, *G* Gabon, *M* Mozambique, *K* Kenya

A total of 1714 pregnant women, 946 (55%) HIV-uninfected and 768 (45%) HIV-infected, were included in this study (Table 1 and Additional file 1: Figure S1). The 3428 peripheral and placental filter papers from these 946 HIV-uninfected and 768 HIV-infected women were tested for the presence and density of *P. falciparum* by qPCR [25, 28]. Covariates at recruitment were similar in the study subgroup and the rest of the women participating in the randomised trial [19, 20] (Additional file 1: Table S1). Prevalence of qPCR-detected *P. falciparum* at delivery, either in peripheral blood at delivery or placental blood (maternal qPCR infection in Table 2), was 41% (143/349) in Benin, 11% (28/257) in Gabon and 6% (21/340) in Mozambique (*P* < 0.001) among HIV-uninfected women, and 9% (32/351) in Kenya and 4% (15/417) in Mozambique (*P* < 0.001) among HIV-infected women (Fig. 1a and Table 2). Similar trends were observed for maternal microscopic infections (Fig. 1b and Table 2). The relationship between microscopy positivity and qPCR parasitaemia in the different study sites is presented in Additional file 1 (Table S2). The proportion of samples that were negative by microscopy but for which qPCR indicated a parasitaemia above 200 parasites/μL was not different between sites (Additional file 1: Table S3).

**Table 2:** Parasitological outcomes in peripheral blood and placenta by study area

	HIV-uninfected				HIV-infected		
	Benin	Gabon	Mozambique	*P*	Kenya	Mozambique	*P*
Periphery at delivery							
Smear, n (%)							
Negative	320 (92)	245 (96)	333 (98)	0.002^a^	332 (95)	408 (98)	0.025^a^
Positive	27 (8)	11 (4)	7 (2)		17 (5)	8 (2)	
qPCR, n (%)							
Negative	242 (69)	235 (91)	325 (96)	<0.001^a^	326 (93)	405 (97)	0.007^a^
Positive	107 (31)	22 (9)	15 (4)		25 (7)	12 (3)	
qPCR parasites/μL, GM (SD)	280.5 (882.9)	684.2 (1703.2)	514.4 (1630.6)	0.484	141.4 (329.1)	3350.9 (9887.2)	0.003
Placenta							
Smear, n (%)							
Negative	322 (93)	244 (96)	332 (98)	0.018^a^	335 (95)	406 (98)	0.032^a^
Positive	23 (7)	9 (4)	8 (2)		16 (5)	7 (2)	
Histology, n (%)							
Acute	8 (2)	5 (2)	1 (0)	<0.001	6 (2)	4 (1)	<0.001
Chronic	23 (7)	2 (1)	2 (1)		5 (1)	1 (0)	
Past	37 (11)	28 (11)	13 (4)		64 (18)	14 (3)	
No infected	277 (80)	218 (86)	322 (95)		276 (79)	387 (95)	
qPCR, n (%)							
Negative	253 (72)	235 (91)	323 (95)	<0.001^a^	324 (92)	407 (98)	0.001^a^
Positive	96 (28)	22 (9)	17 (5)		27 (8)	10 (2)	
qPCR parasites/μL, GM (SD)	668.5 (2473.1)	1284.9 (5100.0)	1876.2 (5910.5)	0.609	379.5 (1255.4)	5851.3 (19950.4)	0.033
Maternal microcopy infection,^b^ n (%)							
Negative	294 (87)	239 (95)	329 (97)	<0.001^a^	325 (93)	396 (98)	0.002^a^
Positive	45 (13)	12 (5)	9 (3)		24 (7)	9 (2)	
Maternal qPCR infection,^b^ n (%)							
Negative	206 (59)	229 (89)	319 (94)	<0.001^a^	319 (91)	402 (96)	<0.001^a^
Positive	143 (41)	28 (11)	21 (6)		32 (9)	15 (4)	

^a^The *P* value is significant according to multivariate analysis adjusted for type of IPTp drug, season, age, gravidity, gestational age, anaemia, literacy, RPR result and MUAC at recruitment, as well as CD4 + T cell count in the case of HIV-positive women (Wald test) (ND for placental histology)

^b^Maternal infections were considered microscopic if *P. falciparum* parasites were observed in peripheral blood at delivery or in placental blood from the maternal side either by microscopy or histology and qPCR infections if peripheral blood at delivery or placental blood samples were positive by qPCR

*GM* geometric mean, *SD* standard deviation

### Effects of *P. falciparum* infection on health outcomes

**Fig. 2:**
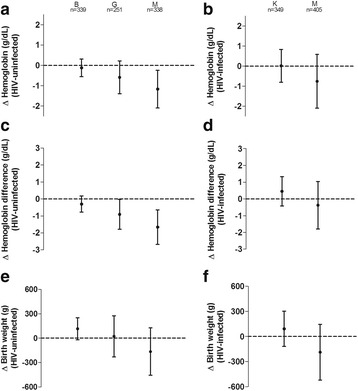
Impact of microscopic malaria infections at delivery on pregnancy outcomes. Maternal microscopic infections were considered present if *P. falciparum* parasites were observed in peripheral blood at delivery and/or in the placenta either by microscopy or histology. The dot and T bar represents the mean difference and 95% confidence interval in haemoglobin levels at delivery (**a**, **b**), the difference of haemoglobin levels from recruitment to delivery (**c**, **d**), or birth weight (**e**, **f**) between malaria infected and uninfected women in the multivariate regression analysis adjusted for type of IPTp drug, season, age, gravidity, gestational age, anaemia, literacy, RPR result and MUAC at recruitment, plus CD4 + T cell count at recruitment in the case of HIV-infected women (**b**, **d**, **f**). Modification of the associations by study area (*B* Benin, *G* Gabon, *K* Kenya, *M* Mozambique) was determined through the inclusion of an interaction term in the regression models, and combination of the coefficients plus the interaction and the standard error was estimated by the delta method

Microscopic *P. falciparum* infection detected at delivery in peripheral blood and/or the placenta of Mozambican women was associated with a reduction of 1.17 g/dL (95% CI –2.09 to –0.24, *P* = 0.014) in their haemoglobin levels, but this reduction was not observed among Beninese nor Gabonese women (*P* = 0.581 and *P* = 0.154, respectively; Fig. 2a). Microscopic infections were also associated with larger drops in haemoglobin levels from recruitment to delivery in Mozambican (–1.66 g/dL, 95% CI –2.68 to –0.64, *P* = 0.001) and Gabonese (–0.91 g/dL, 95% CI –1.79 to –0.02, *P* = 0.045) women, but not among Beninese women (*P* = 0.213; Fig. 2c). No impact of microscopic *P. falciparum* infection in the mother was observed on the birthweight, irrespective of study area (Fig. 2e). However, past or chronic placental *P. falciparum* infection, defined by the presence of *P. falciparum* pigment (i.e. hemozoin) on placental histologic examination, was associated with a reduction of 247.2 g (95% CI –479.1 to –15.2, *P* = 0.037) in the birthweight of Mozambican babies, which was not observed in Benin (*P* = 0.569) and Gabon (*P* = 0.486). Given the strong association between birthweight and gestational age at delivery (Additional file 1: Table S4), the regression model assessing the relationship between birthweight and infection was adjusted by preterm birth, showing a loss of statistical significance (*P* = 0.192). However, past or chronic placental *P. falciparum* infection were associated with an increased risk of preterm birth in Mozambican newborns (OR = 7.05, 95% CI 1.79 to 27.82, *P* = 0.005), but not among Beninese (*P* = 0.785) and Gabonese newborns (*P* = 0.639). Microscopic *P. falciparum* infection was not associated with reductions in haemoglobin levels of HIV-infected women from Kenya and Mozambique nor with reductions of birthweight (Fig. 2b, d, f). Finally, no adverse clinical impact was observed for *P. falciparum* submicroscopic infections, defined by the detection of *P. falciparum* by qPCR but not by microscopy, irrespective of the HIV-infectious status of the pregnant women (Additional file 1: Figure S2). Adjusting variables that remained in the final regression models and interactions assessed with parity, age and IPTp treatment are detailed in Table S5 and S6 of Additional file 1.

### Submicroscopic *P. falciparum* infections, antimalarial immunity and HRP2 levels

**Fig. 3:**
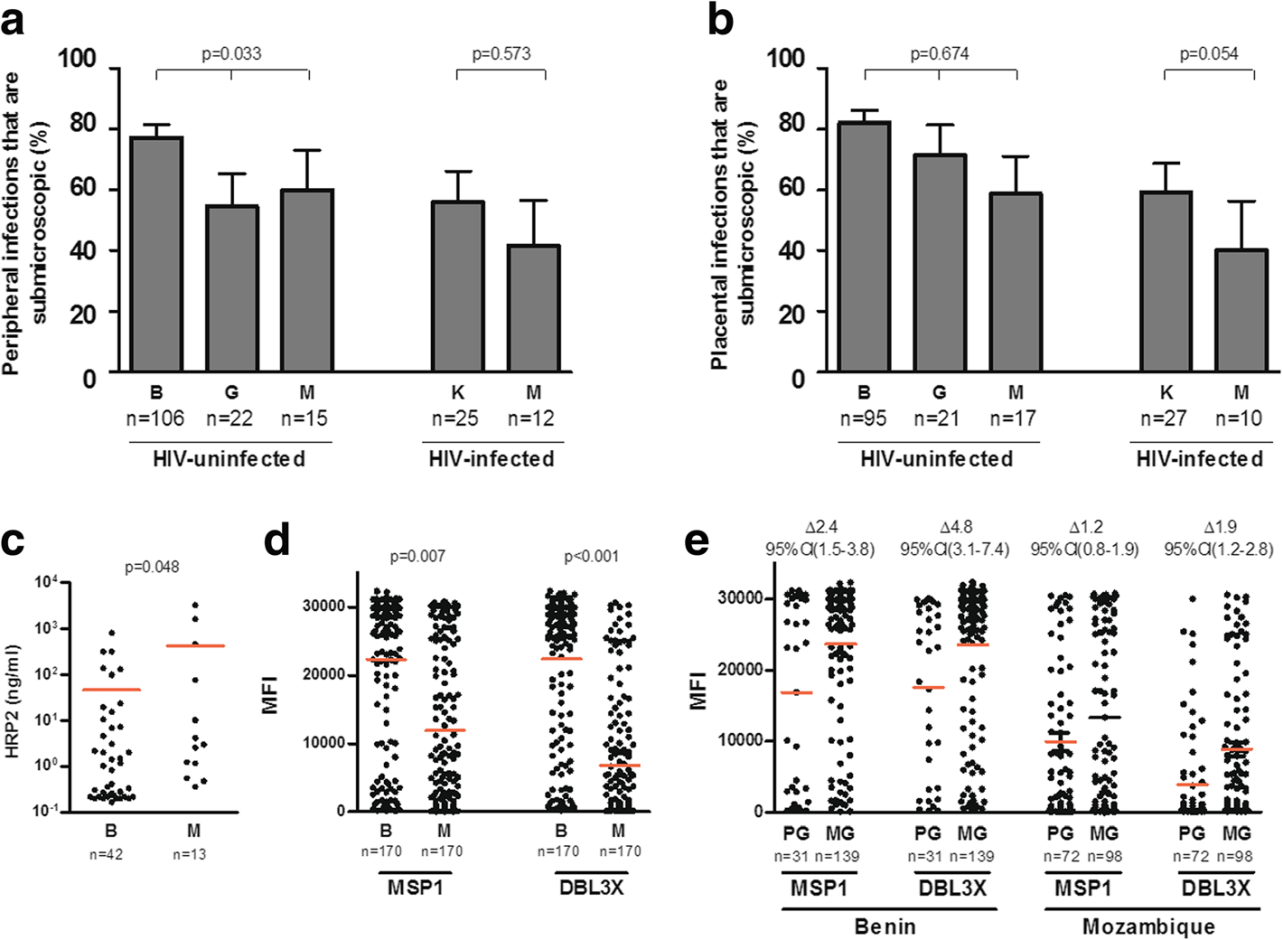
Proportion of *P. falciparum* submicroscopic infections, PfHRP2 concentrations and antibody responses against *P. falciparum* antigens according to study area. Proportion of infections that are submicroscopic in peripheral (**a**) and placental (**b**) blood. *P* value according to the multivariate analysis adjusted for type of IPTp drug, season, age, gravidity, gestational age, anaemia, literacy, RPR result and MUAC at recruitment, as well as CD4 + T cell count in the case of HIV-infected women. T bars represent standard errors. *B* Benin, *G* Gabon, *M* Mozambique, *K* Kenya. **c** PfHRP2 concentrations, indicative of overall parasite biomass, among women with a peripheral qPCR-detected infection in Mozambique (*M*; n = 13) and Benin (*B*; n = 42). **d** Levels of IgG antibodies against MSP1 and DBL3X in Beninese (n = 170) and Mozambican (n = 170) women. **e** Levels of IgG antibodies against MSP1 and DBL3X by parity (primigravidae (*PG*) versus multigravidae (*MG*)), with the proportional increase (Δ) of the antibody levels plus 95% confidence interval in multigravidae versus primigravidae women. *P* values obtained from regression analysis adjusted for type of IPTp drug, season, gravidity, gestational age, anaemia, literacy, RPR result and MUAC at recruitment, as well as CD4 + T cell count in the case of HIV-infected women. *MFI* mean fluorescence intensity

The proportion of peripheral submicroscopic infections among HIV-uninfected women was the highest in Benin (83%; 82/106), followed by Mozambique (60%, 9/15) and Gabon (55%, 12/22, *P*_adjusted_ = 0.033; Fig. 3a). Similar trends, although not statistically significant, were observed for placental submicroscopic infections (82% (78/95), 58% (10/17) and 71% (15/21), respectively, *P*_adjusted_ = 0.674; Fig. 3b). Among pregnant women with a qPCR-detected infection either in peripheral or placental blood, the proportion of submicroscopic infections in any of the two compartments was the highest in Benin (94%, 124/132), followed by Gabon (82%, 18/22) and Mozambique (74%, 14/19, *P*_adjusted_ = 0.028). PfHRP2 concentrations, indicative of overall parasite biomass [26], were higher in pregnant women with a peripheral qPCR-detected infection from Mozambique (n = 13) than from Benin (n = 42, *P* = 0.048; Fig. 3c). HRP2 levels decreased with parity of pregnant women in Benin (0.13, 95% CI 0.02 to 0.79, *P* = 0.028) but not in Mozambique (1.09, 95% CI 0.09 to 12.21, *P* = 0.945). In contrast, age was not associated with differences in HRP2 levels among Beninese (*P* = 0.052) and Mozambican women (*P* = 0.123). Levels of IgGs against DBL3X from VAR2CSA and MSP1 were higher in women from Benin than in women from Mozambique (*P* < 0.007; Fig. 3d). IgG responses against MSP1 increased with parity of Beninese pregnant women (2.39, 95% CI 1.49 to 3.83, *P* < 0.001) but not among Mozambican pregnant women (*P* = 0.401). IgG responses against DBL3x increased with parity of Beninese and Mozambican pregnant women although the magnitude of the increase was higher among Beninese (4.77, 95% CI 3.08 to 7.38, *P* < 0.001) than Mozambican women (1.86, 95% CI 1.23 to 2.79, *P* = 0.003; Fig. 3e). Age was not associated with differences in IG responses irrespectively of antigen and study area (*P* = 0.223 for MSP1 and *P* = 0.171 for DBL3X in Benin and *P* = 0.959 for MSP1 and *P* = 0.380 for DBL3X in Mozambique). No differences in covariates at recruitment were observed between the women with plasma available for serological tests and the rest of the women. The proportion of submicroscopic infections did not differ among HIV-infected women from Kenya and Mozambique (Fig. 3a, b).

### *P. falciparum* densities and pregnancy outcomes

**Fig. 4:**
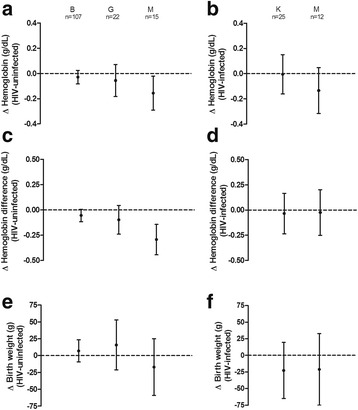
Relationship of qPCR-detected parasite densities in peripheral blood with pregnancy outcomes. The dot and T bar represent the effect and 95% confidence interval on haemoglobin levels at delivery (**a**, **b**), the difference of haemoglobin levels from recruitment to delivery (**c**, **d**), or birth weight (**e**, **f**) due to a two-fold increase in the parasitemia levels in its natural scale. Modification of the associations by study area (*B* Benin, *G* Gabon, *K* Kenya, *M* Mozambique) was determined through the inclusion of an interaction term in the regression models, and combination of the coefficients plus the interaction and the standard error was estimated by the delta method. Analyses were adjusted for type of IPTp drug, season, age, gravidity, gestational age, anaemia, literacy, RPR result and MUAC at recruitment, as well as CD4 + T cell count in the case of HIV-infected women (**b**, **d**, **f**)

Doubling qPCR parasite densities in peripheral blood of HIV-uninfected Mozambican women was associated with a reduction of 0.16 g/dL (95% CI –0.29 to –0.02, *P* = 0.023) in their haemoglobin levels. In contrast, haemoglobin levels remained unaffected by increasing parasite densities in Beninese (*P* = 0.287) and Gabonese women (*P* = 0.381; Fig. 4a and Additional file 1: Figure S3). Similarly, doubling qPCR parasite densities in peripheral blood of HIV-uninfected Mozambican women was associated with a larger drop in the haemoglobin levels from recruitment to delivery (–0.29 g/dL, 95% CI –0.44 to –0.14, *P* < 0.001), while no relationship was found in Beninese (*P* = 0.076) and Gabonese women (*P* = 0.176; Fig. 4c and Additional file 1: Figure S3). No association was found between qPCR parasite densities and birthweight irrespectively of the country of women, nor with pregnancy outcomes in HIV-infected women (Fig. 4).

## Discussion

Although the importance of tolerance in the context of *P. falciparum* infection was suggested early on [29], the link between such a host defence strategy and malaria disease has been obscured by the difficulties of quantifying the tolerance phenotype. We sought to reduce the complexity of tolerance to a single metric based on the relationship between parasite densities and health outcomes (haemoglobin levels and birthweight) [8], with a flat slope indicative of tolerance to infection. To assess for variations in the levels of resistance to *P. falciparum* among women from the different endemic areas, we used the proportion of submicroscopic infections at delivery and IgG levels against parasite antigens as an indicator of the ability of pregnant women to restrict parasite growth [1]. Here, we have demonstrated that the lowest levels of immune resistance and tolerance observed among HIV-uninfected Mozambican women, compared to women from Benin, were accompanied by the largest adverse impacts of *P. falciparum* infections. Thus, the reduced severity of infections observed in pregnant women from high endemic regions [1] may not be mediated entirely by an adaptive immune response to the parasite, but also by a tolerance to *P. falciparum*, as quantified by the slope of the relationship between parasite densities and haemoglobin levels [8]. Taken together, these results provide evidence that pregnant women develop exposure-dependent mechanisms to minimise malaria pathology which, in concert with immune resistance, can reduce the adverse impact of *P. falciparum* infections.

Variation in resistance to *P. falciparum* among HIV-uninfected women from three sub-Saharan African countries was suggested by different proportions of submicroscopic infections. Prevalence of qPCR-detected *P. falciparum* infections at delivery ranged from 41% in Benin to 11% in Gabon and 6% in Mozambique. Pregnant women from Benin had the highest proportion of submicroscopic infections at delivery, suggesting an increased capacity to maintain infections at densities bellow the detection limit of microscopy. These observations are in contrast with trends reported showing a significantly higher percentage of infections detected by microscopy in the general population residing in areas of high compared to those in low transmission areas [30]. These discrepancies may suggest a special dynamic and progression of *P. falciparum* infection during pregnancy compared to infections in non-pregnant hosts. In line with this, PfHPR2 levels in plasma, indicative of total parasite biomass [26], were lower among Beninese than Mozambican women, whereas levels of anti-parasite antibodies, as well as the increase with parity of IgGs against DBL3X from VAR2CSA, were higher in women from Benin than from Mozambique. Overall, these data supports the notion that the acquisition of antimalarial immunity after exposure to *P. falciparum* parasites can increase the capacity to resist *P. falciparum* growth during subsequent infections in pregnancy.

Variations in tolerance to *P. falciparum* were also observed in pregnant women living under contrasting levels of malaria transmission, as indicated by differences in the relationship between haemoglobin levels and increasing parasite burden. In this study, haemoglobin levels at delivery decreased as parasite densities increased in HIV-uninfected women from the lowest transmission setting in Mozambique. By contrast, the haemoglobin level in HIV-uninfected Beninese and Gabonese women were not affected by parasite density, indicating a better tolerance to the infection [7]. Parasite factors such as the level of resistance to SP, as well as the protective effect of IPTp with SP against adverse birth outcomes that are related to curable sexually transmitted and reproductive tract infections [31, 32], may affect the clinical impact of infections among pregnant women who received antimalarials as IPTp. However, no relationship was observed between the level of molecular markers of SP resistance in the parasite population based on the frequencies of dihydropteroate synthase (*Pfdhps*) K540E mutation [33, 34] previously reported (>90% *Pfdhps*-K540E in Kenya [33] but < 50% in Mozambique [35], Gabon [36] and Benin [37]) and the outcomes of the study. Moreover, the efficacy of IPTp with SP to clear peripheral parasites and prevent new infections during pregnancy has been suggested to be compromised only in areas with > 90% prevalence of *Pfdhps* K540E mutation [33]. Taken together, these results suggest that immunoregulatory responses that reduce pathogenic inflammation and potentially the risk of anaemia [38] may be developed by pregnant women exposed to *P. falciparum*, as has been suggested for children [39–41].

In absence of HIV infection, the adverse clinical impact of *P. falciparum* infections was the highest in pregnant women from the low transmission site in Mozambique, who had the lowest levels of immune resistance and tolerance to *P. falciparum*. This adverse impact was observed for microscopic *P. falciparum* infections, which were associated with reductions in maternal haemoglobin levels at delivery as well as with increased drops in haemoglobin levels from recruitment to delivery, but not with birthweight. Moreover, placental past/chronic infections among Mozambican women were associated with an increased risk of preterm births, in accordance with previous studies showing a larger impact of infections during pregnancy compared to infections detected only at delivery [42–44]. Such an adverse effect of placental past/chronic infections on the birthweight of newborns was not observed among Beninese or Gabonese women. *P. falciparum* microscopic infection in Gabonese women was also associated with an increased drop in haemoglobin levels from recruitment to delivery, although of a lower magnitude than the drop observed in Mozambican women. These data suggest that the malaria-related adverse impact in the health of pregnant women is higher in Gabon than in Benin, but lower than in Mozambique. Such an intermediate severity of the infections in Gabonese women might be explained by the development of tolerance to *P. falciparum* (as suggested by the lack of an association between haemoglobin levels with increasing parasite densities) rather than by immune resistance to the infection (as indicated by the similar carriage of submicroscopic infections in Gabonese and Mozambican women). Overall, this evidence suggests that resistance and tolerance to malaria can be acquired after exposure to *P. falciparum* parasites in areas of high transmission, and can reduce the detrimental consequences of *P. falciparum* infections. Importantly, HIV-infected pregnant women from Kenya and Mozambique did not show any evidence of varying levels of resistance or tolerance, suggesting that the ability to limit the adverse impact of *P. falciparum* infection may be reduced when the immune system is suppressed by the viral infection [45].

This study has some limitations. First, *P. falciparum* infection at delivery in women who received IPTp most likely reflect a recently acquired infection. Thus, this study may under-estimate the adverse impact of *P. falciparum* infections during pregnancy, as compared to recent reports showing that submicroscopic *P. falciparum* infection at inclusion (16.5 weeks) increases the risk of low birth weight for primigravid and premature delivery for multigravid pregnant Beninese women [6]. Second, other factors apart from anti-parasite immunity may contribute to the carriage of submicroscopic infections, such as the stage of the infection, the chronicity of infections, which is most common among primigravid women without immunity to placental parasites [46], and the existence of suppressive levels of antimalarial drugs. Third, site-specific differences in the extent of healthcare provided and economic development, as well as other factors than can affect pregnancy outcomes (i.e. haemoglobinopathies), might contribute to variations in the clinical impact of infections among pregnant women from different countries. However, the impact of these differences was minimised by the fact that this study was performed in the context of a clinical trial following standard procedures [19, 20] and that the analysis was adjusted by potential confounders. Fourth, the immunological analyses were conducted only in a subset of plasma samples available from the women included in the study, which were similar in covariates with the rest of the women. Fifth, analyses were performed by group, rather than at the individual level, assuming uniform exposure and risk among all individuals at each site. Finally, the small prevalence of *P. falciparum* infection among HIV-positive women who were receiving co-trimoxazole during pregnancy [20] may have reduced the power to detect variations in resistance and tolerance to malaria.

## Conclusions

This study shows that pregnant women may reduce the deleterious impact of *P. falciparum* infection through two conceptually different types of defence against malaria, namely, one that targets the parasite (resistance), and the other that prevents the damage induced by the infection (tolerance) [7, 8]. Down-regulation of malaria-induced inflammation may mediate this tolerance phenotype, although other mechanisms that increase the resilience to tissue injuries, such as metabolic adaptation to tissue repair and detoxification of pathogen by-products [11, 47], might still be involved. Making the distinction between resistance mechanisms that restrict parasite multiplication from those that minimise the harm caused by the infection [7, 8] is important to understand how reductions in transmission intensity can affect host defences and, therefore, the clinical presentation of *P. falciparum* infections, as well as for the design of new host-directed therapies [9, 48, 49].

## Additional file

Additional file 1: Supplementary Tables and Figures. (DOCX 279 kb)

## Abbreviations

DBL3X: Duffy-Binding Like 3 X
HRP2: Histidine-Rich Protein2
IPTp: intermittent preventive treatment during pregnancy
MQ: mefloquine
MSP1: Merozoite Surfce Protein 1
*P. falciparum*: *Plasmodium falciparum*
qPCR: quantitative polymerase chain-reaction
SP: sulfadoxine-pyrimethamine
