# Supplementary material for: Resisting and tolerating P. falciparum in pregnancy under different malaria transmission intensities
Source: BMC Med. 2017 Jul 17;15:130. doi: 10.1186/s12916-017-0893-6 (PMC5513247; doi:10.1186/s12916-017-0893-6)
Supplement: Additional file 1: — Supplementary Tables and Figures. (DOCX 279 kb) [file 12916_2017_893_MOESM1_ESM.docx]

**ADDITIONAL MATERIAL**

**Resisting and tolerating *P. falciparum* in pregnancy under different malaria transmission intensities**

**Table S1.** Characteristics of the women participating in the intermittent preventive treatment trials and those not included in the study.

**Table S2.** Relationship between microscopy positivity and qPCR parasitaemia in the different study sites.

**Table S3.** Submicroscopic infections by qPCR parasite density (lower or higher than 200 parasites/µl) and study site.

**Table S4.** Relationship between gestational age at delivery (assessed by Ballard score) and pregnancy outcomes. a) describes the relationship assessed by Spearman correlation and b) the comparison of outcomes between normal and preterm births.

**a**

**b**

**Table S5.** Adjusting variables that remained in the final regression models.

**Table S6.** Interactions with parity, age and IPTp treatment in regression models assessing the relationship between pregnancy outcomes and *P. falciparum* infection.

The modification of the associations by parity, age and IPTp treatment was assessed by including interaction terms into the regression models and combining the coefficients plus the interaction and the standard error by the delta method.

**Figure S1.** Study profile.

**
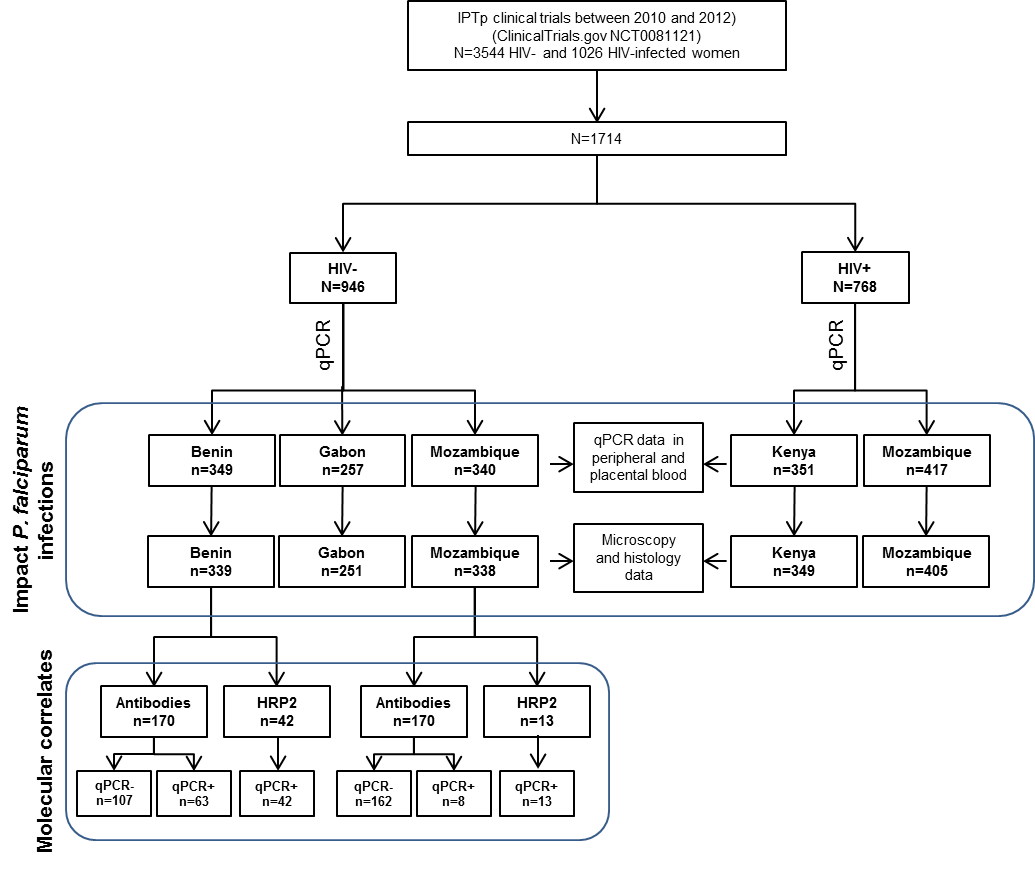
**

**Figure S2.** Impact of malaria submicroscopic infections in peripheral blood on pregnancy outcomes.

Submicroscopic infections were defined as *P. falciparum* infections detected by qPCR but negative by microscopy. The dot and T bar represents the mean difference and 95% confidence interval in hemoglobin levels (**a, d**), the difference of haemoglobin levels from recruitment to delivery (**b, e**), or birth weight (**c, f**) between malaria infected and uninfected women in the multivariate regression analysis adjusted for type of IPTp drug, season, age, gravidity, gestational age, anemia, literacy, RPR result and MUAC at recruitment, plus CD4+T cell count at recruitment in the case of HV-infected women (**d, e, f**). Modification of the associations by study area (B=Benin, G=Gabon, K=Kenya, M=Mozambique) was determined through the inclusion of an interaction term in the regression models, and combination of the coefficients plus the interaction and the standard error was estimated by the delta method.

**Figure S3.** Relationship between parasite densities in peripheral blood measured by qPCR and haemoglobin levels and the difference in haemoglobin levels from recruitment to delivery (Δ Hemoglobin) in HIV-uninfected women from Benin (**a, d**), Gabon (**b, e**) and Mozambique (**c, f**). Solid red lines indicate best fit.
